# Single‐nucleotide polymorphisms in 3′‐untranslated region inducible costimulator gene and the important roles of miRNA in alopecia areata

**DOI:** 10.1002/ski2.34

**Published:** 2021-05-06

**Authors:** G. Conteduca, A. Rossi, F. Megiorni, A. Parodi, F. Ferrera, S. Tardito, T. Altosole, V. Fausti, C. Occella, F. Kalli, S. Negrini, A. Pizzuti, C. Marchese, E. Rizza, F. Indiveri, D. Coviello, D. Fenoglio, G. Filaci

**Affiliations:** ^1^ Laboratory of Human Genetics IRCCS Istituto Giannina Gaslini Genoa Italy; ^2^ Department of Anesthesiology and Cardiovascular Clinical Internal Sciences “Sapienza” University of Rome Rome Italy; ^3^ Department of Experimental Medicine “Sapienza” University of Rome Rome Italy; ^4^ Biotherapies Unit IRCCS Ospedale Policlinico San Martino Genoa Italy; ^5^ Centre of Excellence for Biomedical Research and Department of Internal Medicine University of Genoa Genoa Italy; ^6^ Dermatology Unit IRCCS Istituto Giannina Gaslini Genoa Italy; ^7^ Associazione Nazionale Alopecia Areata Genoa Italy

## Abstract

**Background:**

Alopecia areata (AA) spares the stem cell compartment and attacks only the base of the hair follicle, which is surrounded by infiltrating lymphocytes. AA is associated with polymorphisms in immune‐related genes and with decreased function of CD4+CD25+ T regulatory (Treg) cells. Treg function is modulated by the costimulatory molecules, like inducible costimulator (ICOS) that are crucial in orienting T cell differentiation and function so that they strongly impact on the immunologic decision between tolerance or autoimmunity development.

**Objective:**

The aim of our study was to investigate the possible association of AA with single‐nucleotide polymorphisms (SNP) present in the ICOS 3′‐untranslated region (3′UTR) region and to elucidate how SNPs modulate ICOS gene expression by affecting miRNA binding sites.

**Methods:**

This is a case‐control study performed in 184 patients with AA and 200 controls. ICOS gene and miRNA expression were analyzed by real‐time polymerase chain reaction.

**Results:**

The genotype carrying the rs4404254(C) [*p* = 0.012, OR (95% CI): 0.5 (0.3–0.8)] and rs4675379(C) [*p* = 0.015, OR (95% CI): 0.3 (0.1–0.8)] 3′ UTR alleles was more frequently observed in AA patients than in controls and correlated with a reduced ICOS expression. miR‐1276 significantly suppressed ICOS expression by binding to the 3′UTR of ICOS mRNA. Also, we observed that, miR‐101 and miR‐27b are upregulated, while miR‐103 and miR‐2355‐3p are downregulated in peripheral blood mononuclear cells of AA patients compared to controls;

**Conclusion:**

Our data show that rs4404254 and rs4675379 SNPs of ICOS gene are associated with AA and also reveal that the presence of rs4404254 polymorphism correlates with ICOS post‐transcriptional repression by microRNA binding.

## Graphical abstract

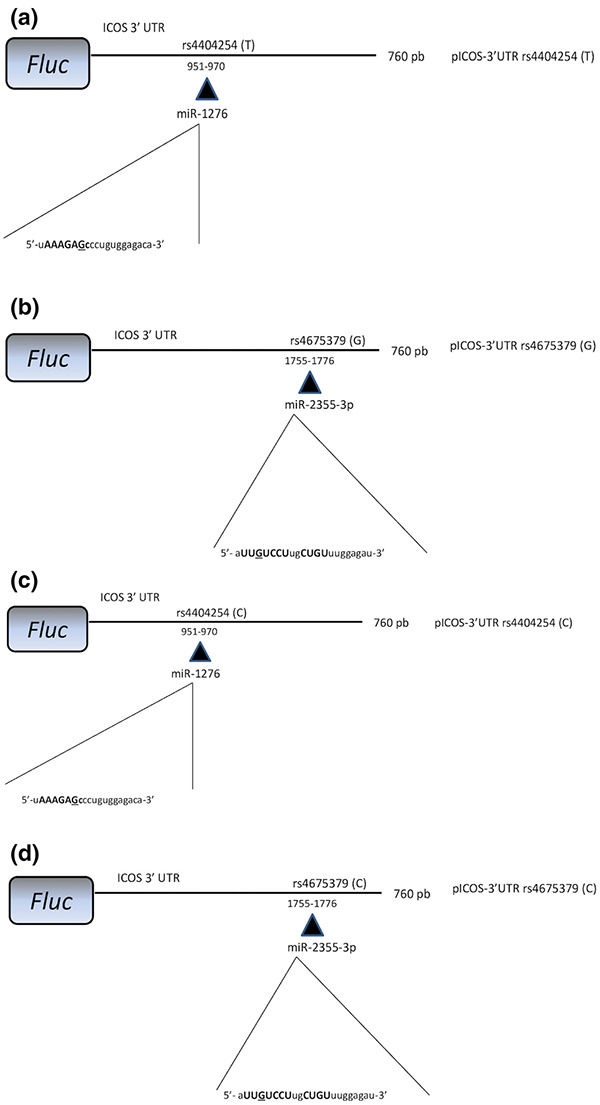

Our data show that rs4404254 and rs4675379 SNPs of ICOS gene are associated with Alopecia Areata and also reveal that the presence of rs4404254 polymorphism correlates with ICOS post‐transcriptional repression by microRNA binding. These results suggest that reduced expression of ICOS in T regulatory cells is relevant to the development of AA.

1

**What's already known about this topic?**

- Alopecia reata (AA) is believed to have an autoimmune pathogenesis by which the hair follicles are targeted by CD4+ and CD8+ T lymphocytes.
- CD4+CD25+ T regulatory cells (Treg) are significantly lower in AA when compared to other cutaneous diseases.
- Several studies demonstrated an association between Alopecia Areata and polymorphisms of immune‐related genes including: (a) HLA molecules; (b) ICOSL; (c) FOXP3; (d) NOTCH4; (d) CTLA4; (e) IKZF4.

**What does this study add?**

- Two single nucleotide polymorphisms (SNPs), rs4404254 and rs4675379, located in the 3′ UTR of inducible costimulator (ICOS) gene are associated with Alopecia areata
- The presence of these polymorphisms correlates with ICOS post‐transcriptional repression by microRNA binding of miR‐101 and miR‐27b which are upregulated, while miR‐103 and miR‐2355‐3p are downregulated in peripheral blood mononuclear cells (PBMCs) of AA patients compared to controls;
- ICOS is downregulated in PBMCs of AA patient compared to healthy controls

## 2 INTRODUCTION

Alopecia areata (AA) affects about 1%–2% of the general population, including males and females across all ethnic groups, with a lifetime risk of 1.7%.^1^, ^2^ Autoimmunity develops against the hair follicle, resulting in non‐scarring hair loss that may begin as patches that can coalesce and progress to cover the entire scalp (alopecia totalis) or eventually the entire body (alopecia universalis). The genetic basis of AA is largely unknown. Genome‐wide association study (GWAS) shows an association with genomic regions containing several genes controlling the activation and proliferation of regulatory T cells (Treg cells), cytotoxic T lymphocyte‐associated antigen 4 (CTLA4), interleukin (IL)‐2/IL‐21, IL‐2 receptor A (IL‐2RA; CD25) and Eos (also known as Ikaros family zinc finger 4; IKZF4), as well as the human leukocyte antigen (HLA) region.^3^ Regulatory T lymphocytes (Treg) are involved in the control of immune homeostasis by preventing autoimmune diseases.^4^ Accordingly, in the C3H/HeJ mouse model for AA and in Alopecia patients Treg alterations have been observed in the skin and in blood.^5^, ^6^ Evidence supporting a genetic basis for AA stems from multiple lines of research, including the observed heritability in first‐degree relatives.^7^, ^8^ More works have shown genetic association in different ethnic groups for AA.^9^, ^10^

Inducible costimulator (ICOS) is a co‐stimulatory receptor involved in activation and function of T lymphocytes.^11^ Interestingly, ICOS‐ICOS‐ligand (ICOSL) interaction has been related to activation and expansion of Treg.^12^, ^13^ We previously showed that rs378299‐509(C) allelic variant in the promoter region of ICOSL gene was associated with reduced relative gene expression in AA patients.^14^ Here, we explored the possibility that five specific single‐nucleotide polymorphisms (SNPs) in the 3′‐untraslated region (3′UTR) of the ICOS gene, selected in the European population (HapMap SNPs by UCSC), could be associated with AA development. We chose to study these SNPs in the 3′UTR, previously also correlated with susceptibility to colorectal cancer,^15^ because this is the region of ICOS gene preferentially bound by miRNAs.

Post‐transcriptional regulation has a key role for innate and adaptive immune responses.^16^, ^17^ One class of transacting factors are microRNAs (miRNAs) that, once loaded onto the RNA‐induced silencing complex, can base pair with partially complementary sequences in the 3′UTR of target mRNAs to silence gene expression.^18^, ^19^

In recent years, gene‐based therapies of hair follicles, including treatments with antisense oligonucleotides, small interfering RNAs and miRNAs, have been proposed in the treatment of hair follicle‐associated diseases and also in male pattern baldness.^20^, ^21^, ^22^ Based on this evidence, we analyzed the expression of miR‐101, miR‐103, miR‐27b and miR‐369 involved in Treg and cytotoxic T cell (TC) differentiation. Also, we correlated miR‐2355‐3p and miR‐1276 (probably implicated in ICOS post‐transcriptional regulation) with the frequency of the ICOS SNP located in the 3′UTR.

## 3 METHODS

### 3.1 Patients and controls

One hundred eighty‐four patients affected with AA (56 males and 128 females, age ranging from 7 to 60 years, 82 with patchy AA, 60 with universalis AA and 42 with totalis AA), and 200 healthy controls (106 males and 94 females, age ranging from 15 to 75 years) from the same ethnic area were analyzed in a case‐control study. Patients were recruited by the Department of Internal Medicine, La Sapienza University, Rome, and among those affiliated to the Associazione Nazionale Alopecia Areata (ANAA onlus), located in Genoa. The diagnosis of AA was performed according to specific criteria.^23^ As controls, we enrolled healthy individuals who had not been affected by any autoimmune disease. The study was approved by the IRCCS Giannina Gaslini ethics committee (Approval no. OCCFAUIGG001201) and have been performed in accordance with the 1964 Declaration of Helsinki and its later amendments. Informed consent was obtained from each participant.

### 3.2 Power calculation

We estimated that a sample size of 184 patients and 200 unmatched controls gives a power of 80% in detecting a difference of allelic frequency between cases and controls from 5% to 15% at a significance level of 5%.

### 3.3 Gene sequencing

DNA was extracted from the peripheral blood cells by the DNA Blood Extraction Kit (Qiagen). The sequences, containing the rs4404254 (T23816C), rs73991306(C23707T), rs10932036 (A23711T), rs77882417 (A23877G), rs10932038 (A24114G), rs1559931 (A24259G), rs10932037(C21876T), rs4675379 (G24625C) SNPs, were polymerase chain reaction (PCR) amplified using primers specifically designed to cover the 3′UTR of ICOS gene (GenBank accession no. AF488347). PCR conditions were optimized using various magnesium concentrations and annealing temperatures. Genomic DNA (100 ng) was used in each PCR reaction. PCR products were purified from primers by the UltraClean PCR Clean‐up Sample Kit (CABRU), automatically sequenced using the ABI BigDye Terminator Ready Reaction Mix (Applied Biosystems) and analyzed by an ABI 3130XL Genetic Analyzer (Applied Biosystems) according to the manufacturer's protocol.

### 3.4 In silico analysis

In order to evaluate the SNP impact on miRNA binding sites in the 3′‐UTR of ICOS mRNA, we performed in silico analysis. Validated target gene of differentially expressed miRNAs were predicted using different bioinformatics tools as already described.^24^ Briefly UCSC (http://genome.ucsc.edu/, hg19 assembly), NCBI (http://www.ncbi.nlm.nih.gov/) and UTRdb (http://utrdb.ba.itb. cnr. it/) genome browsers provided information about the human ICOS gene. In order to identify putative miRNAs targeting ICOS 3′UTR, we performed in silico analysis by the common prediction algorithms TargetScan (http://www.targetscan.org, release 7.1), miRBase (http://microrna.sanger.ac.uk, release 22.1), miRanda (http://www. microrna.org), miRecords (http://mirecords.biolead.org/), miRNA SNIPER (https://tools4mirs.org), miRNA SNP V1.0 (http://www.bioguo.org, release 2.0) and miRWalk 2.0 (http://mirwalk.umm.uni‐heidelberg.de).

These web servers use algorithms searching for target sequences with perfect or nearly perfect pairing to the 3′UTR sequence, evaluating the thermodynamic stability of miRNA‐mRNA hybrids and performing comparative sequence analysis to check evolutionary conservation.

### 3.5 Expression analysis of ICOS and miRNAs in AA PBMCs

For ICOS and miRNA expression analysis, peripheral blood mononuclear cells (PBMCs) were isolated by standard Ficoll‐Hypaque density centrifugation (Biochrom) from 30 patients and 30 healthy controls.

ICOS gene expression was analyzed by PCR and real‐time PCR as follows. Total RNA of 100 ng, isolated using the OMNIZOL RNA Isolation Kit (EuroClone), were incubated with 6 U DNase I and reverse transcribed into cDNA using Oligo (dT) 20Primer and Superscript II Reverse Transcriptase (Invitrogen), followed by RNase H digestion. RNA integrity was assessed using Agilent Bioanalyzer 2100 (Agilent Technologies Inc.) and quantified with a NanoDrop‐2000 spectrophotometer (Thermo Fisher Scientific). RNA integrity number (RIN) values were ranging from 7.5 to 10.0. We performed the following PCR conditions in a Thermalcycler (Eppendorf): a single denaturation step at 94°C for 3 min followed by 35 cycles at 94°C for 1 min, then 62°C for 1 min and 72°C for 2 min, followed by a final extension step at 72°C for 10 min. The following oligonucleotide pairs were used (sense and antisense, respectively): ICOS, 5' ‐TCTGGCACCCAGGCATGAAG‐3′ and 5′‐TGCTTTGCAGATTCAGTACC‐3'; GAPDH, 5′‐GAGCAACAGGAAGTGGCTGTG‐3′ and 5′‐TAATGCTTCCAGTTTACAAGTGGT‐3'. Expression levels of ICOS gene and selected miRNAs were evaluated by SYBR green Master Mix (Roche Diagnostics, Ltd.) with the above‐described primers. The values of cDNA expression were normalized to glyceraldehyde‐3‐phosphate dehydrogenase (GAPDH) expression. miRNA expression analysis was performed as previously reported.^25^, ^26^ Briefly, stem‐loop primers, designed to have a short single‐stranded part that is complementary to an anchor primer, were employed to quantify miRNA expression. The anchor RT primer was used as the template for the negative control reaction and U6 small nuclear RNA was used as a control to determine relative miRNA expression. The 2^‐∆∆ C*t*^ method formula was used as described.^27^ The primers used are listed in Table S1.

### 3.6 Site‐directed mutagenesis and luciferase assay

Three x10^5^ 293T cells were plated into 24‐well plates in Roswell Park Memorial Institute (RPMI) medium supplemented with 10% fetal bovine serum (FBS) 24 h prior to transfection. The 3′UTR region of ICOS gene (965–1774 bp of 3′ region) containing the predicted target sites of miR‐1276 and miR‐2355‐3p or mutated ICOS 3′UTR were cloned into the pGL3‐Report luciferase vector (Promega, E1751) as described.^24^ Site‐directed mutagenesis was performed with Taq DNA Polymerase Kit (Thermo Fisher Scientific) and specific designed primers: ICOS for 2M 5′‐ACAAGTTTAGCTCTTTTTGTAGATC‐3′, ICOS rev2M 5′‐GATCTACAAAAAGAGCTAAACTTGA‐3′, ICOS for3M 5′‐GGGTATGGGGAGGACAACCTT‐3′, ICOS rev3M 5′‐AAGGTTGTCCTCCCCATACCC‐3′, ICOS XbaI for 5′‐GCTCTAGAGACAGCCCAACAGCCACTCT‐3′, ICOS XbaI rev1 5′‐GCTCTAGACCTTGGGCATGCAGACAGGAAGTA‐3′, ICOS XbaI for3 5′‐GCTCTAGAATTTTTGCTCCAGAAAGACATGTTC‐3′, ICOS XbaI rev3 5′‐GCTCTAGATGTCGAGGCACAGCTTGGAC‐3'.

293T cells were co‐transfected, using DOTAP (Roche) as vehicle, with two different pICOS‐3′UTR rs4404254 plasmids containing ICOS variant or wild‐type alleles, respectively, or two pICOS‐3′UTR rs4675379 plasmids bearing ICOS variant or wild type alleles, respectively, and with 50nM of either the miR‐1276 or miR‐2355‐3p mimic, or a miRNA negative control (Qiagen). Cells were harvested after 48 h and luciferase protein production was analyzed by Firefly luciferase FITC conjugate antibody (Abcam) in flow cytometry assays. All assays were performed in triplicate and the experiments were repeated three times. All the luciferase data were expressed as the mean fluorescence normalized to the negative control for the same reporter construct.

### 3.7 Cell lines

293T embryonic kidney cell line was purchased from ATCC (ATCC CRL‐3216^TM^). Cells were grown at 37°C in a 5% CO_2_ atmosphere in RPMI‐1640 medium (SIGMA) supplemented with 10% FCS, 2 mM L‐glutamine, 10 mmol HEPES, 100 mmol non‐essential amino acids, 100 U/ml penicillin, 100 μg/ml streptomycine and 1% sodium pyruvate (referred to as culture medium).

### 3.8 Flow cytometry

For staining of intracellular luciferase, cells were fixed with citofix/cytoperm (Becton Dickinson Bioscience) at 4°C for 20 min, rinsed twice with permeabilization buffer (PBS, 1% FBS, 0.1% saponin), and incubated with a luciferase specific FITC‐conjugated mAb (100ng/1 × 10^5^cells) (Abcam ab21176). After incubation cells were resuspended in staining buffer to be then analyzed by flow cytometry using a FACSCanto instrument (Becton Dickinson Biosciences). Isotype and subclass matched goat IgG (100 ng/ml) were used as negative controls in all the experiments (Figure S1). Cell Quest software (Becton Dickinson Biosciences) was used for data analysis.

### 3.9 Statistical analysis

Statistically significant differences between genotype frequencies were assessed using univariate or multivariate analyses such as the Fisher's exact test for binary variables and the Student *t* test for continuous variables. Odds ratio (ORs) and 95% confidence intervals (CIs) were calculated and *p* values lower than 0.05 were considered statistically significant. Analyses were performed using SPSS 13.0 and Graphpad Prism 4 softwares. A test for the Hardy–Weinberg equilibrium was performed by GenAlEx 6 software. Because multiple pair wise tests are performed on a single set of data, taking into account variables such as age, sex and alopecia subtypes, a Bonferroni‐adjusted significance level of 0.05 was calculated to account for the increased possibility of obtaining false‐positive results.

## 4 RESULTS

### 4.1 Frequency of ICOS 3′UTR SNPs in AA patients compared with controls

Genotypic and allelic frequencies of rs4404254, rs73991306, rs10932036, rs77882417, rs10932038, rs1559931, rs10932037 and rs4675379 SNPs, located in the 3′UTR of the ICOS gene, were analyzed in AA patients and in healthy controls. The distribution of the genotypes resulted in a Hardy–Weinberg equilibrium in both patients and controls (Table S2). The distribution of genotypes of the studied polymorphisms in AA patients and controls is shown in Table 1. Statistically significant differences were observed in the distributions of ICOS rs4404254 *T*  >  C and ICOS rs4675379 G> C genotypes between controls and AA patients (*p* = 0.012 and *p* = 0.015, respectively) (Table 1). No association was observed between rs4404254 or rs4675379 SNPs and sex or AA stage. No difference was observed in the distribution of others ICOS 3′UTR SNPs between AA patients and controls.

**TABLE 1:** Comparison of the frequency of ICOS UTR3′ SNPs between alopecia patients and controls

Genotypes	Percent frequency in controls/patients	OR (95% CI)	*p*
rs4404254 variant genotypes^a^ (TC + CC)	19/29	0.5 (0.3–0.8)	0.012
rs73991306 variant genotypes (CT + TT)	9/6	1.5 (0.7–3.5)	0.25
rs10932036 variant genotypes (AT + TT)	0.5/0.5	0.9 (0.05–14.8)	0.95
rs77882417 variant genotypes (AG + GG)	0.5/0.5	0.9 (0.05–14.8)	0.95
rs10932038 variant genotypes (AG + GG)	4/1	4.7 (0.9–24.9)	0.063
rs1559931 variant genotypes (AG + GG)	1/4	0.3 (0.7–1.2)	0.091
rs10932037 variant genotypes (CT + TT)	1/2	0.7 (0.1–3.3)	0.66
rs4675379 variant genotypes^a^ (GC + CC)	4/11	0.3 (0.1–0.8)	0.015

Abbreviations: ICOS, inducible costimulator; OR, odd ratio; SNPs, single‐nucleotide polymorphisms.

^a^ The C variant of the rs4404254 SNP and the C variant of the rs4675379 SNP are a risk factor for the disease. OR was calculated for the frequency of the minor variant allele in control versus patients.

### 4.2 rs4404254(C) and rs4675379(C) 3′UTR alleles correlate with reduced ICOS gene expression

**FIGURE 1:**
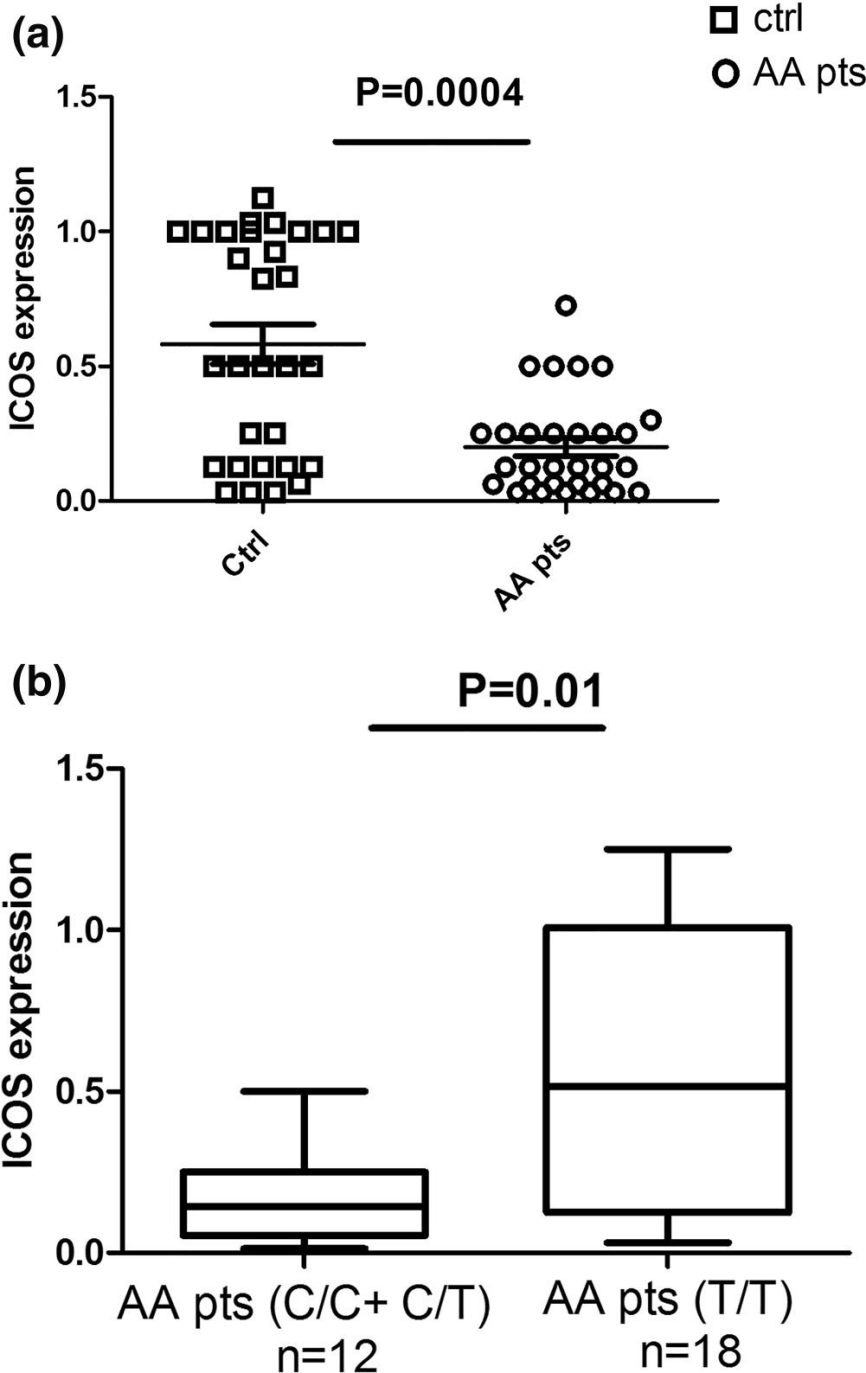
The risk allele of rs4404254 is associated with lower inducible costimulator (ICOS) mRNA expression. (a) Relative expression measurements of ICOS expression were calculated by real‐time PCR. Fold change (*y*‐axis) represents the relative expression of ICOS in 30 AA patients (AA pts) in comparison to 30 healthy controls (ctrl) (*p* = 0.0004). (b) Correlation analysis between the SNP genotypes and ICOS expression in 12 AA patients carrying the rs4404254 risk allele, and 18 patients carrying the rs4404254 non‐risk allele. For the rs4404254 risk allele we considered the C/C and C/T genotypes, and for the non‐risk allele the T/T genotype (*p* = 0.01). Error bars in (a) and (b) represent mean and *SD* of three independent experiments. Normalization of the expression data for the ICOS gene was realized with GAPDH using the 2^‐∆∆ C*t*^ method formula

Since rs4404254 and rs4675379 SNPs are located in the 3′UTR of the ICOS gene, it is reasonable to hypothesize that they could modulate its relative gene expression. Hence, we analyzed ICOS mRNA levels in PBMCs of AA patients (AA pts) and controls (ctrl). Also, we have compared ICOS expression levels in blood between different subset cohorts of AA patients carrying several variant alleles in DNA for rs4404254 or rs4675379 SNP. The intention of this analysis was to analyze a potential effect of the AA‐associated SNPs with ICOS expression levels. We compared AA patients bearing the ICOS risk alleles with AA patients bearing the non‐risk alleles. We considered for the rs4404254(C) risk allele patients with the C/C or C/T genotypes, and for the rs4675379(C) risk allele we analyzed patients with the C/C or C/G genotypes. AA patients showed lower levels of ICOS gene expression compared to healthy subjects (Figure 1a) (*p* = 0.0004). However, concerning the rs4404254 SNP, AA patients, homozygotes or heterozygotes for the C allele, had lower ICOS mRNA levels than those carrying the T/T homozygous allele (Figure 1b). Concerning the rs4675379 SNP, ICOS gene expression was not significantly different in patients carrying the (C) allele (C/C + C/G) with respect to those carrying only the reciprocal (G) allele (Figure S2).

### 4.3 miRNA quantification in PBMC

We analyzed the mean relative expression of six selected miRNAs (miR‐101, miR‐103, and miR‐27b, miR‐369, miR‐2355‐3p and miR‐1276), which have been reported to be involved in the differentiation pathways of T lymphocytes and in the development of autoimmune diseases.^28^, ^29^, ^30^ Interestingly, different studies have already described that miR‐101, miR‐103, and miR‐27b can bind 3′UTR region of ICOS mRNA.^31^, ^32^, ^33^ Moreover, computational interrogation (using miRNA SNP V1.0 server) of miRNA responsive elements (MREs) within the ICOS 3′UTR predicted a good binding (as indicated by the obtained score values) of miR‐369, miR‐2355‐3p and miR‐1276 to ICOS 3′UTR mRNA depending on rs4404254 and rs4675379 SNPs.

**FIGURE 2:**
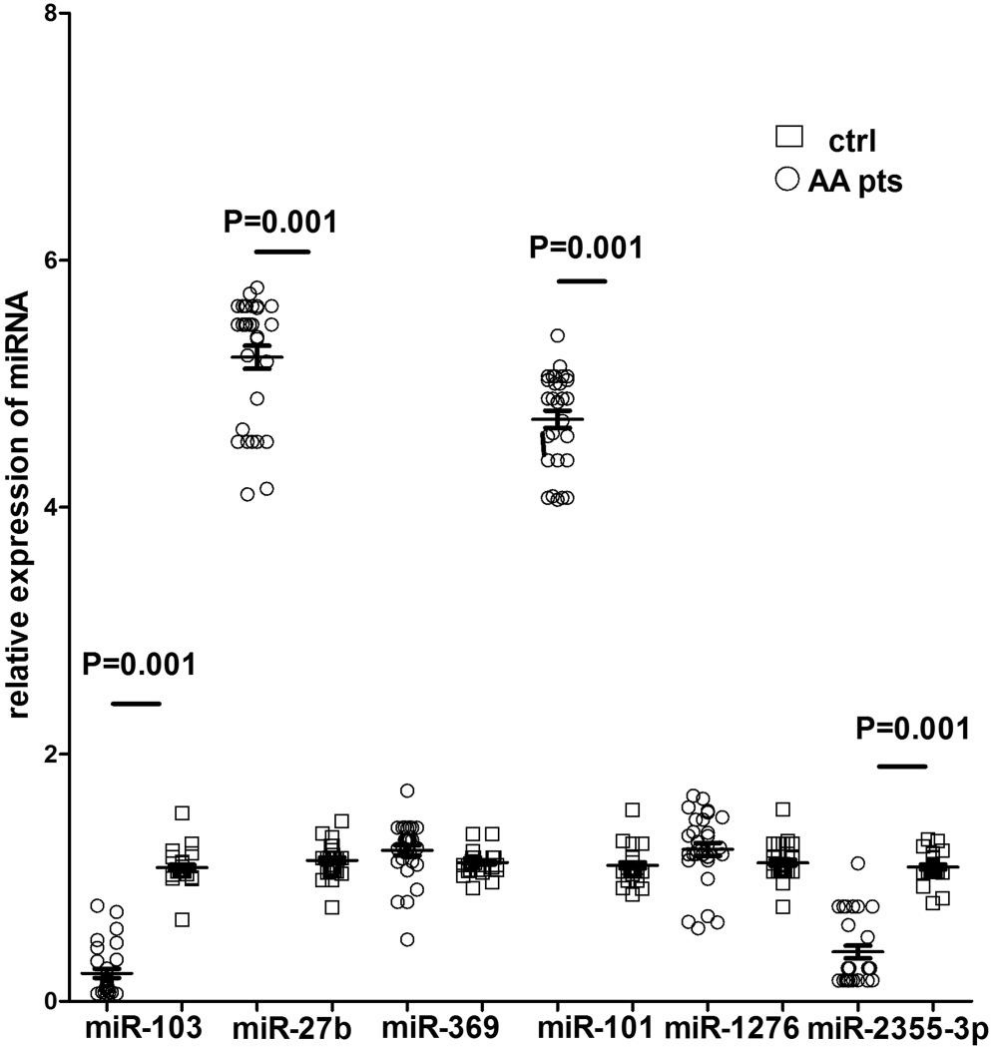
Relative expression of miRNA in PBMCs from healthy subjects and AA patients. Relative expression measurements of miRNA were calculated by real‐time PCR, using the 2^‐∆∆ C*t*^ method formula. Fold change (*y*‐axis) represents the relative expression of the miR‐103, miR‐27b, miR‐369, miR‐101, miR‐1276 and miR‐2355‐3p genes in AA patients (circular plots) in comparison to healthy controls (rectangular plots). U6 small nuclear RNA was used as a control to determine relative miRNA expression. Error bars represent mean and *SD* of three independent experiments

The mean relative expression of these miRNA in PBMCs of 30 different individuals with AA and in 30 healthy controls are shown in Figure 2. In particular, miR‐101 and miR‐27b were significantly upregulated, with mean fold changes of 4.5 (*p* < 0.001), and 5.27 (*p* < 0.001), respectively, in AA patients relative to healthy controls. miR‐103 and miR‐2355‐3p were significantly downregulated in AA PBMCs, with mean fold changes of 0.12 (*p* < 0.001), and 0.15 (*p* < 0.001), respectively, in patients with respect to controls (Figure 2). No significant differences in the expression of miR‐369, and miR‐1276 were observed in PBMCs of AA patients and healthy controls.

### 4.4 Post‐transcriptional regulation of ICOS expression by miR‐2355‐3p and miR‐1276 specific binding

After miRNA expression analysis, we performed in silico analysis, in order to evaluate if the effect of miRNA on post‐transcriptional regulation of ICOS gene was influenced by rs4404254 or rs4675379 SNPs. By computational prediction of MREs within the 3′UTR of ICOS mRNA, we found 210 putative miRNAs able to post‐transcriptionally regulate ICOS expression. The most likely miRNA candidates targeting the ICOS 3′UTR bearing rs4404254 and rs4675379 SNPs resulted miR‐2355‐3p and miR‐1276. This result was obtained by overlapping the outputs of the miRNA‐based prediction programs SNIPER, miRecords and miRNA SNP V1.0 (Table S3). Indeed, we selected miR‐2355‐3p and miR‐1276 miRNAs as the more likely regulators of the ICOS mRNA by combining the results of the prediction programs with thermodynamic and on‐line available expression data (Table 2). In particular, miR‐2355‐3p responsive element is an 8‐mer, whilst miR‐1276 target site is a 6‐mer element that shares an exact complementarity to position 2–5 of the mature miRNA. The rs4404254 polymorphism concerns a *T* > C nucleotide substitution which results in a change from a G:T mismatch to a G:C pair between ICOS 3′UTR and miR‐1276 hybrid structure, while rs4675379 SNP concerns a G > C nucleotide substitution which results in a change from a G:G mismatch to a G:C pair between ICOS 3′UTR and miR‐2355‐3p hybrid structure. We found that these changes from a G:T or G:G mismatch to a G:C pair impact on the free energy of the hybrid. In fact, the miR‐2355‐3p:ICOS mRNA heteroduplex displayed a free energy value of −12.10 kcal/mol for rs4675379 (G) wild type allele and −21.20 kcal/mol for rs4675379 (C) variant allele; whereas miR‐1276:ICOS hybrid structure showed a −15.40 kcal/mol value for rs4404254(T) wild‐type allele and −17.80 kcal/mol value for rs4404254(C) variant allele (Table 2). Thermodynamic stability of the miRNA‐mRNA duplex is given by the free energy gained by binding of miRNA to the target site and is denoted by ΔG. Thermodynamic accessibility has also been argued to be an important predictor of miRNA repression^34^: therefore, we evaluated free energy value of the miRNA:ICOS mRNA heteroduplex.

**TABLE 2:** Bioinformatic prediction of microRNAs potentially targeting the ICOS gene

SNP in gene3′UTR	miRNA	SNP location and target site on UTR	Chromosome position of gene target	Energy change (kcal/mol)	miRNA/SNP‐target duplexes	Effect by SNP on 3′UTR
ICOS rs4404254 (U/C)	Has‐miR1276	964951‐970	Gene NM_012092 chr2:204,821,369 ‐ 204,821,408 (on assembly GRCh37)	Wild:‐15.40SNP:‐17.80^a^	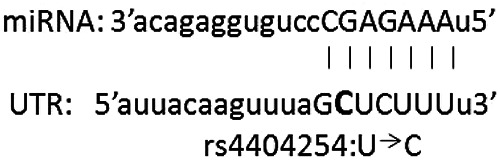	Gain
ICOS rs4675379 (G/C)	Has‐miR2355‐3p	17731755‐1776	Gene NM_012092 chr2:204 821 369 ‐ 204 821 408 (on assembly GRCh37)	Wild:‐12.10SNP:‐21.20^a^	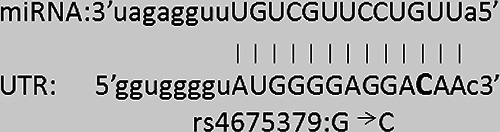	Gain

Abbreviations: ICOS, inducible costimulator; SNPs, single‐nucleotide polymorphisms; 3′UTR, 3′‐untranslated.

^a^ Numbers indicate the predicted miR‐1276 and miR‐2355‐3p seed sequences using the numbering of the ICOS 3′UTR and predicted ICOS 3′UTR hybrid structure with miR‐1276 or miR‐2355‐3p and mean free energy (kcal/mol) obtained by miRNA SNP V1.0 server.

**FIGURE 3:**
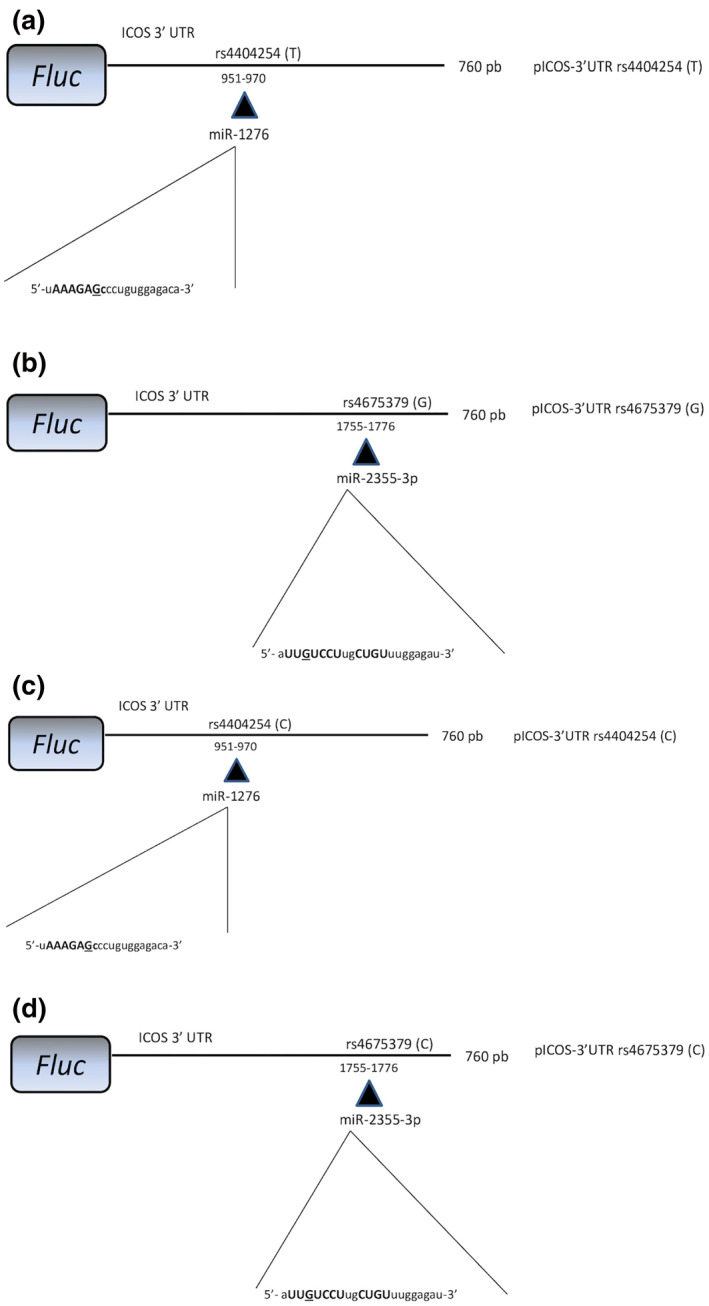
Schematic representation of the construct used in the luciferase assays. A fragment of 760 bp of the ICOS 3′UTR, encompassing rs4404254 wild‐type allele (a) or rs4404254 allelic variant (c) the putative responsive elements for miR‐1276 or encompassing rs4675379 wild type (b) or rs4675379 allelic variant (d), the putative responsive elements for miR‐2355‐3p, was cloned in pGL3 vector downstream to the Firefly luciferase coding sequence. Nucleotides in miRNA sequence paired with ICOS 3′UTR are shown in capital letters and miRNA nucleotide paired with the corresponding allelic variant of ICOS 3′UTR SNPs is underlined in bold

To verify whether miR‐2355‐3p and/or miR‐1276 were able to target ICOS expression, we performed in vitro experiments. 293T cells were co‐transfected either with a reporter construct containing 760 base pairs of the human ICOS 3′UTR downstream of the Firefly luciferase open reading frame, or with a control Firefly plasmid, together with wild type rs4404254(T) or rs4675379(G) SNP (for miR‐1276 or for miR‐2355‐3p, respectively) (Figure 3a,b), or with their relative rs4404254(C) or rs4675379(C) variant alleles (Figure 3c,d).

**FIGURE 4:**
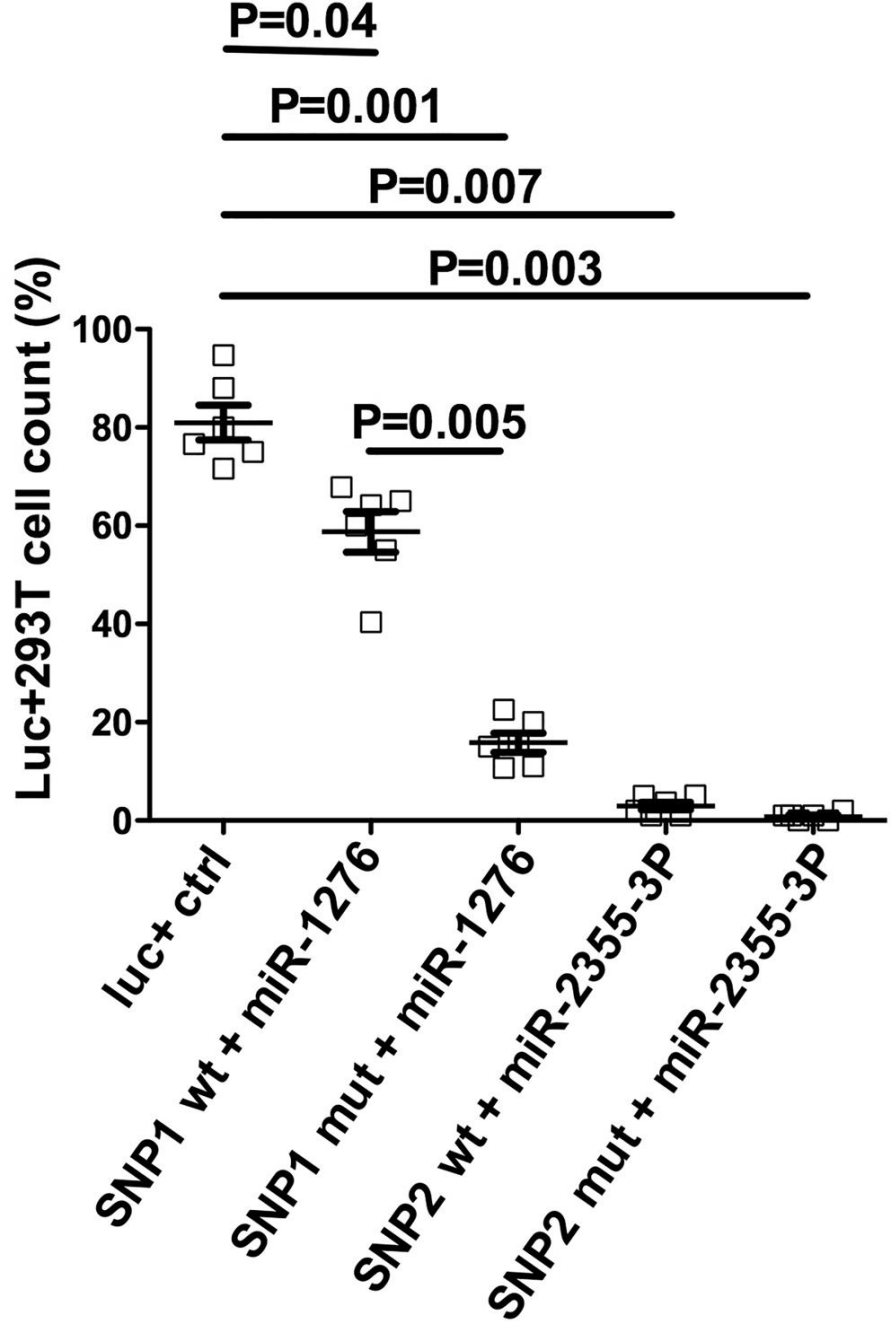
miR‐1276 and miR‐2355‐3p target the ICOS 3′UTR. 293T cells were transfected independently with pGL3‐Report luciferase plasmid or pICOS‐3′UTR vector together with either specific microRNA miR‐1276 or miR‐2355‐3p. Forty‐eight hours after transfection, the percentage of cells expressing the protein luciferase was measured by flow cytometry and normalized to the Firefly control. The results represent the mean and standard error (SE) of at least three independent experiments, each carried out in triplicate. The risk allele for rs4404254 SNP was (C) allelic variant (SNP1 mut) and the non‐risk allele was (T) allelic variant (SNP1 wt), while for rs4675379 SNP the risk allele for was (C) allelic variant (SNP2 mut), and the non‐risk allele was (G) allelic variant (SNP2 wt)

Both miR‐1276 and miR‐2355‐3p significantly suppressed luciferase expression compared to positive reporter construct containing Firefly luciferase (Figure 4). In particular, decrease in Firefly luciferase expression of about 65% (*p* = 0.001) and 80% (*p* = 0.007) for rs4404254(C) and rs4675379(C) variant alleles, respectively, was observed (Figure 4). miR‐1276 was also able to inhibit Firefly luciferase expression by pICOS‐3′UTR rs4404254(T) wild‐type allele (24%, *p* = 0.04) and by pICOS‐3′UTR rs4404254(C) ICOS variant allele (41%, *p* = 0.001) (Figure 4). No significant differences were evident when 293T cells were co‐transfected with miR‐2355‐3p and with pICOS‐3′UTR rs4675379(C) bearing variant or pICOS‐3′UTR rs4675379(G) containing wild=‐type allele (*p* > 0.05) (Figure 4). These data suggest that the presence of the rs4675379(C) risk alleles into the specific miRNA binding‐sites in the ICOS 3′UTR is able to reduce the inhibitory function of miR‐1276 and miR‐2355‐3p. Thus, they indicate that miR‐1276 and miR‐2355‐3p functionally interact with the ICOS 3′‐UTR and suppress the translation of the corresponding protein product.

## 5 DISCUSSION

Strong evidences suggest that genetic predisposition may contribute to AA development.^3^, ^35^ The aim of the present study was to investigate the association of the ICOS SNPs in the 3′UTR with the susceptibility to AA in an Italian case series of patients. Also, because the 3′UTR is the principal binding site for miRNAs, we wanted to study the correlation between AA and miRNA gene expression that were influenced by an associated SNP.

The present study improves our knowledge on the genetic variants predisposing to AA and the important roles of miRNAs. The results of the study show that rs4404254(C) and rs4675379(C) allelic variants in the ICOS 3′UTR are significantly more frequent in patients affected with AA than in healthy controls (*p* < 0.05). Also, rs4404254(C) allelic variant is associated with a reduced expression of ICOS genes in AA patients. Because ICOS regulates the generation and function of Treg,^13^ these data confirm that polymorphisms in the 3′UTR of ICOS gene may alter gene expression, possibly impacting on Treg activity in AA. Indeed, our data indicate an association between the rs4404254(C) and rs4675379 (C) allelic variants of the ICOS gene with AA. Interestingly, in AA patients the presence of these alleles was related to a reduced gene expression, event that functionally may determine an altered activation of Treg. Many works show the involvement of miRNAs in hair biology, and in AA disease.^21^, ^36^ In AA, different miRNA expression levels contribute to the establishment of the inflammatory, angiogenesis, proliferative responses and they are involved in AA.^37^ miRNAs also have been shown to be critical in the maintenance of Treg stability that is required for the preservation of suppressor function during inflammation that can occur in AA.^38^ ^,^ ^39^ Accordingly, we observed that miR‐101 and miR‐27b are upregulated, while miR‐103 and miR‐2355‐3p are downregulated in PBMCs of AA patients compared to controls. In the last decade, many studies have shown that SNP in 3′UTR regions, which are known to contribute to mRNA stability and localization, as well as gene translational efficiency,^40^ may result in a 90% decrease of ICOS expression, that, in turn, may induce impairment of Treg functions.^41^ Interestingly, different studies showed correlation between ICOS rs4404254 or rs4675379 and ICOS expression in different tumours, where ICOS rs4404254(C) allele and rs4675379(C) are associated with tumour risk.^41^ In the present work, we studied the effects of ICOS 3′ UTR related polymorphisms, rs4404254 and rs4675379 on ICOS post‐transcriptional regulation by miR‐1276 and miR‐2355‐3p. We performed in vitro analysis by luciferase assay, in order to verify in silico prediction results on miRNA and ICOS SNP interaction.

In conclusion, based on the current results, rs4404254 is a functional SNP in ICOS and the different alleles of the SNP can alter the ability of miR‐1276 to bind and degrade ICOS mRNA in vitro. The present study improves our knowledge on the genetic variants predisposing to AA and the important roles of miRNAs. Therefore, dysregulated miRNAs may be important diagnostic markers and therapeutic targets for patients. These results suggest that some new aspects for treatment of AA could be revealed. Stability of ICOS expression and suppressive function are of paramount concern for Treg therapies in AA inflammatory environment and would be amenable to miRNA manipulation. Similar Treg therapies are being used to treat diabetes^42^ ^,^ ^43^ and could be used to restore normal expression of miRNA in Treg.

Indeed, studies in other series of patients are needed to verify whether our results apply to AA patients of different ethnicity.

## CONFLICT OF INTERESTS

The authors declare that there are no conflict of interests.

## Supporting information

Supplementary Material

## Data Availability

The data that support the findings of this study are available in the supplementary material of this article.
