# Supplementary material for: Single‐nucleotide polymorphisms in 3′‐untranslated region inducible costimulator gene and the important roles of miRNA in alopecia areata
Source: Skin Health Dis. 2021 May 6;1(2):e34. doi: 10.1002/ski2.34 (PMC9060044; doi:10.1002/ski2.34)
Supplement: Supplementary file 1 — Supplementary Material [file SKI2-1-e34-s001.doc]

| **Table 1. Primers used for miRNA expression analysis** | |
| --- | --- |
| U6-forward primer | 5’CTCGCTTCGGCAGCACA3’ |
| U6-reverse primer | 5’AACGCTTCACGAATTTGCGT3’ |
| Universal reverse primer (URP) | 5′TCGTGGAGTCGGCAA3′ |
| miR-101- forward primer | 5’TGGGCTACAGTACTGTGATA3’ |
| miR-103-forward primer | 5’GAGCAGCATTGTACAG3’ |
| miR-27b-forward primer | 5’AGAGCTTAGCTGATTGGTGAAC3’ |
| miR-369-forward primer | 5’GTCCACTAGCCGTCCGTATC 3’ |
| miR-1276-forward primer | 5’TAAAGAGCCCTGTGGAGACA3’ |
| miR-2355-3p forward primer | 5’ATCCCCAGATACAATGGACAA3’ |
| miR-101-Adapter Primer | 5’TGGGCTACAGTACTGTGATAAAAAAAAAATGTCTCGCCTACCACACCCTTACCG**TTGCCGACTCCACGA**3’ |
| miR-103- Adapter Primer | 5’GAGCAGCATTGTACAGAAAAAAAAAATGTCTCGCCTACCACACCCTTACCG**TTGCCGACTCCACGA**3’ |
| miR-27b- Adapter Primer | 5'AGAGCTTAGCTGATTGGTGAACAAAAAAAAAATGTCTCGCCTACCACACCC**TTACCGTTGCCGACTCCACGA**3’ |
| miR-369- Adapter Primer | 5’GTCCACTAGCCGTCCGTATCAAAAAAAAAATGTCTCGCCTACCACACCC**TTACCGTTGCCGACTCCACGA**3’ |
| miR-1276- Adapter Primer | 5’TAAAGAGCCCTGTGGAGACAAAAAAAAAAATGTCTCGCCTACCACACCC**TTACCGTTGCCGACTCCACGA**3’ |
| miR-2355-3p-Adapter Primer | 5’ATCCCCAGATACAATGGACAAAAAAAAAAAATGTCTCGCCTACCACACCC**TTACCGTTGCCGACTCCACGA**3’ |

| **Table 2.** **Genotype distribution for Hardy–Weinberg equilibrium (HWE)** | | |
| --- | --- | --- |
| **Genotypes** | **n (%)** | |
|  | **Patients**  **(n=184)** | **Controls**  **(n=200)** |
| rs4404254 T/C | | |
| T/T | 130 | 162 |
| C/T | 49 | 35 |
| C/C | 5 | 3 |
| HWE | P=0.98^1^ | P=0.78^1^ |
| rs4675379 G/C | | |
| G/G | 163 | 191 |
| C/G | 20 | 9 |
| C/C | 1 | 0 |
| HWE | P=0.91^1^ | P=0.94^1^ |

^1^The p-value is higher than 0.05, suggesting no deviation of HWE

| **Table 3.** **Overlap between the different tools and in vitro luciferase assay results for interaction study between miR1276- ICOS rs4404254 SNP and miR2355-3p- ICOS rs4675379 SNP** | | | | | |
| --- | --- | --- | --- | --- | --- |
|  | miRNA SNP V1.0 server | miRBase | miRanda | TargetScan | in vitro luciferase assay |
| ICOS gene target miR1276 | + | + | + | + | + |
| ICOS gene target miR2355-3p | + | - | + | - | + |
| Energy change  (kcal/mol) score for ICOS rs4404254 SNP: | + | - | + | + | + |
| Energy change  (kcal/mol) score for ICOS rs4675379 SNP: | + | + | - | - | - |
